# Formation and excretion of autophagic plastids (plastolysomes) in *Brassica napus* embryogenic microspores

**DOI:** 10.3389/fpls.2015.00094

**Published:** 2015-02-19

**Authors:** Verónica Parra-Vega, Patricia Corral-Martínez, Alba Rivas-Sendra, Jose M. Seguí-Simarro

**Affiliations:** Cell Biology Group, COMAV Institute, Universitat Politècnica de ValènciaValencia, Spain

**Keywords:** androgenesis, cryomethods, electron microscopy, microspore embryogenesis, rapeseed

## Abstract

The change in developmental fate of microspores reprogrammed toward embryogenesis is a complex but fascinating experimental system where microspores undergo dramatic changes derived from the developmental switch. After 40 years of study of the ultrastructural changes undergone by the induced microspores, many questions are still open. In this work, we analyzed the architecture of DNA-containing organelles such as plastids and mitochondria in samples of *B. napus* isolated microspore cultures covering the different stages before, during, and after the developmental switch. Mitochondria presented a conventional oval or sausage-like morphology for all cell types studied, similar to that found *in vivo* in other cell types from vegetative parts. Similarly, plastids of microspores before induction and of non-induced cells showed conventional architectures. However, approximately 40% of the plastids of embryogenic microspores presented atypical features such as curved profiles, protrusions, and internal compartments filled with cytoplasm. Three-dimensional reconstructions confirmed that these plastids actually engulf cytoplasm regions, isolating them from the rest of the cell. Acid phosphatase activity was found in them, confirming the lytic activity of these organelles. In addition, digested plastid-like structures were found excreted to the apoplast. All these phenomena seemed transient, since microspore-derived embryos (MDEs) showed conventional plastids. Together, these results strongly suggested that under special circumstances, such as those of the androgenic switch, plastids of embryogenic microspores behave as autophagic plastids (*plastolysomes*), engulfing cytoplasm for digestion, and then are excreted out of the cytoplasm as part of a cleaning program necessary for microspores to become embryos.

## Introduction

Microspore embryogenesis is the most efficient biotechnological approach to obtain haploid individuals and doubled haploid (DH) lines. DHs are used as pure lines in breeding programs to produce hybrid seeds. They are also extremely valuable tools for plant genetic research (Forster et al., 2007; Seguí-Simarro, 2010). Microspore embryogenesis consists of the reprogramming of the microspores (the pollen precursors) toward embryogenesis. This developmental switch (also known as androgenesis) is generally induced through the application of stress. The microspores of some species can be induced by starvation, others by the application of cold temperatures to the inflorescences prior to *in vitro* culture, and others by the application of a heat shock to the *in vitro* cultured microspores, as is the case for *Brassica napus* microspores (reviewed in Shariatpanahi et al., 2006). Once microspores are reprogrammed, they undergo multiple changes to readapt themselves to the new developmental scenario. These changes include, among others, a profound remodeling of gene expression, the triggering of a (stress) response as a consequence of the inductive (stressing) treatment, the suppression of the ongoing gametophytic program, and the initiation of embryogenesis (Maraschin et al., 2005; Seguí-Simarro and Nuez, 2008; Dunwell, 2010). At the subcellular level, there is also an extensive remodeling of cell ultrastructure, including a displacement of the nucleus to the center of the cell, a rearrangement of the cytokinetic machinery, a switch from an asymmetric to a symmetric division pattern, and a reduction in the number of plastids (Zaki and Dickinson, 1991; Hause et al., 1993; Telmer et al., 1995; Testillano et al., 2000; Shariatpanahi et al., 2006; Makowska and Oleszczuk, 2014).

The study of the ultrastructural changes associated to the androgenic switch started approximately 40 years ago with the pioneering works of Dunwell and Sunderland (1974a,b, 1975, 1976a,b,c). During these decades, these studies have been traditionally done by using transmission electron microscopy (TEM) in samples preserved with aldehyde-based chemical fixatives. The main disadvantage of these fixatives is the parallel generation of structural disorders in membranous elements of different subcellular compartments and organelles (McDonald and Auer, 2006). Among these artifacts, chemical fixatives may generate membrane retraction, fusion, and/or swelling, as well as vesiculation of large membranous elements (Gilkey and Staehelin, 1986). Such a change of the original cell ultrastructure frequently precludes the accurate identification and analysis of complex membranous structures (Gilkey and Staehelin, 1986; McDonald and Auer, 2006). Fortunately, there is an alternative to avoid the artifacts of chemical fixatives, which consists on the combined use of two cryotechniques for sample preservation: High Pressure Freezing and Freeze Substitution (HPF/FS). HPF consists on freezing the sample within milliseconds while subjected to high pressure (2100 bar). HPF/FS prevents the formation of ice crystals derived from freezing and provides an excellent ultrastructural preservation, much better than chemical fixation (Gilkey and Staehelin, 1986). These features make HPF/FS the method of choice for fine ultrastructural analysis. Using this method, Corral-Martínez et al. (2013) found evidence for the extensive formation of autophagosomes engulfing from small to large regions of cytoplasm, and the occurrence of massive autophagy prior to excretion of the partially digested cytoplasmic material to the apoplast. These were exclusive features of just induced microspores, not present in cells neither before nor long after the inductive stage. It seemed that in induced cells, the autophagosomes and the vacuolar system worked together as a cytoplasmic cleaning mechanism to adapt the cell to a new embryogenic scenario.

In this work, we also applied HPF/FS to study isolated microspore cultures of *B. napus*, in order to find out whether cytoplasmic organelles undergo similar autophagic processes. Indeed, the transformation of initially normal plastids into autophagic compartments has been previously described in plastids of other cell types, including senescing suspensor cells of *Phaseolus coccineus* (Nagl, 1977) and *Phaseolus vulgaris* (Gärtner and Nagl, 1980) and in petal cells of *Dendrobium* (van Doorn et al., 2011). For the present work, different stages of microspore embryogenesis (before and after the inductive treatment) were covered, including vacuolate microspores and pollen grains, both before induction, and induced and non-induced microspores, as well as microspore-derived embryos (MDEs), after the induction. We focused on the analysis of DNA-containing organelles such as plastids and mitochondria, and analyzed their ultrastructure and development during the process of embryogenesis induction and further MDE development. Our results demonstrate that many plastids (but not mitochondria) of embryogenic microspores undergo dramatic structural changes as a consequence of embryogenesis induction. We propose that these changes are related to autophagy and excretion of the engulfed material.

## Materials and methods

### Plant material

*B. napus* L. cv. Topas was used as the donor plants for isolated microspore culture. Donor plants were grown at 20°C under natural light in the greenhouses of the University of Colorado (Boulder, CO, USA) and the COMAV Institute (Universitat Politècnica de València, Valencia, Spain).

### Isolated microspore culture of *brassica napus*

Flower buds from 3.3 to 3.4 mm were collected. The microspores were isolated in NLN-13 medium that consists on NLN medium (Nitsch and Nitsch, 1967) +13% sucrose. Isolated microspore cultures were performed as previously described (Corral-Martínez et al., 2013). Briefly, microspores were isolated and subjected to a heat stress treatment at 32°C for 24 h to induce embryogenesis, and then cultured in darkness at 25°C for progression of embryogenesis. Cultures were monitored on a daily basis under an inverted microscope. Dishes at different stages were collected and processed by HPF/FS as explained below.

### Processing of samples for TEM

Three different types of *B. napus* samples were processed for TEM, including anthers containing microspores and pollen at different stages of microsporogenesis and microgametogenesis, 4-day-old cultured microspores, and MDEs at different developmental stages: globular, heart-shaped and torpedo embryos. All samples were randomly selected and three different sample batches were processed as previously described (Corral-Martínez et al., 2013; Seguí-Simarro, 2015a). Briefly, samples were fixed with a Baltec HPM 010 (Technotrade, Manchester, NH, USA) and a Leica EM HPM 100 (Leica Microsystems, Vienna, Austria) high pressure freezers. After HPF, samples were freeze substituted in a Leica AFS2 system. Substitution was performed with 2% OsO_4_ in anhydrous acetone. Infiltration was carried out with increasing concentrations of Epon resin (Ted Pella, Redding, CA, USA) diluted in acetone, and polymerization was performed at 60°C for 2 days. A minimum of five resin blocks were randomly selected and sectioned for further analysis. A Leica UC6 ultramicrotome was used to obtain thin sections (~1 μm) and ultrathin (~80 nm) sections for observation at light microscopy and TEM, respectively. Ultrathin sections were mounted on formvar and carbon-coated, 200-mesh copper grids, stained with uranyl acetate and lead citrate, observed and photographed in a Philips CM10 TEM. A minimum of 100 electron micrographs were taken and analyzed at each of the stages studies. For the stage presenting atypical plastids, 242 electron micrographs were taken and studied. For the quantitative analysis of atypical plastids, 107 of these images, containing at least one plastid per image, were analyzed.

### FESEM-FIB three-dimensional reconstruction of subcellular volumes

The 3-D study of the plastids and mitochondria contained within the induced microspores was carried out with a FESEM-FIB (Auriga Compact, Zeiss) as described in Seguí-Simarro (2015b). This technique combines a Field Emission Scanning Electron Microscope (FESEM) and a Focused gallium Ion Beam (FIB) to sequentially mill the sample surface. Briefly, the sample-containing part of HPF/FS-processed resin blocks was smoothened with a diamond knife, separated, and placed into the specimen stage of the FESEM-FIB. The areas of interest (embryogenic microspores) were visualized using the secondary electron detector. Then, the sample was tilted to 52°. The ion beam was used to mill a window exposing the areas of interest. Then, the samples were milled with the FIB (operating at 1 nA) removing 20 nm thick layers from the sample surface. Images were acquired every 40 nm. Two different stacks from two different areas of interest were acquired. The images were aligned with MIDAS and the reconstruction was made with 3 dmod, both included in the IMOD software package (Kremer et al., 1996).

### *In situ* detection of acid phosphatase activity

The assay of acid phosphatase activity was performed over ultrathin sections according to Gärtner and Nagl (1980). The nature of this cytochemical reaction imposes the use of hydrated samples and temperatures above 0°C. For these reasons, this assay was performed on chemically fixed specimens, instead of using HPF for fixation. Three day-old *B. napus* microspore cultures were collected and fixed with 6.25% glutaraldehyde and 3% DMSO in 0.05 M cacodylate buffer (pH 7.4) at 4°C for 45 min. After three washes with 0.05 M cacodylate buffer, samples were incubated for 1 h at 37°C in a solution containing 10 ml 0.2 M Tris-maleate buffer (pH 5.2), 6 ml 0.02 M lead nitrate, 4 ml 0.1 M β-glycerophosphate disodium salt, and 30 ml distilled water. Control incubation was made replacing the substrate (β-glycerophosphate) by distilled water. After incubation, the samples were washed three times with 0.04 M Tris-maleate buffer (pH 5.2) at 4°C, 30 min each. Then, samples were postfixed with 1% OsO_4_ in 0.05 M cacodylate buffer for 4 h, and washed three times with same buffer, 30 min each. Samples were dehydrated through increasing series of ethanol at 4°C, and then infiltrated with increasing concentrations of Spurr resin (Ted Pella, Redding, CA) in ethanol. Polimerization was carried out at 70°C for 24 h. Thin (~1 μm) and ultrathin (~80 nm) sections were obtained for light microscopy and TEM, respectively, using a Leica UC6 microtome. Ultrathin sections were mounted on formvar and carbon-coated, 200-mesh copper grids, stained with uranyl acetate and lead citrate, and finally observed and imaged in a Philips CM10 TEM.

## Results

**Figure 1:**
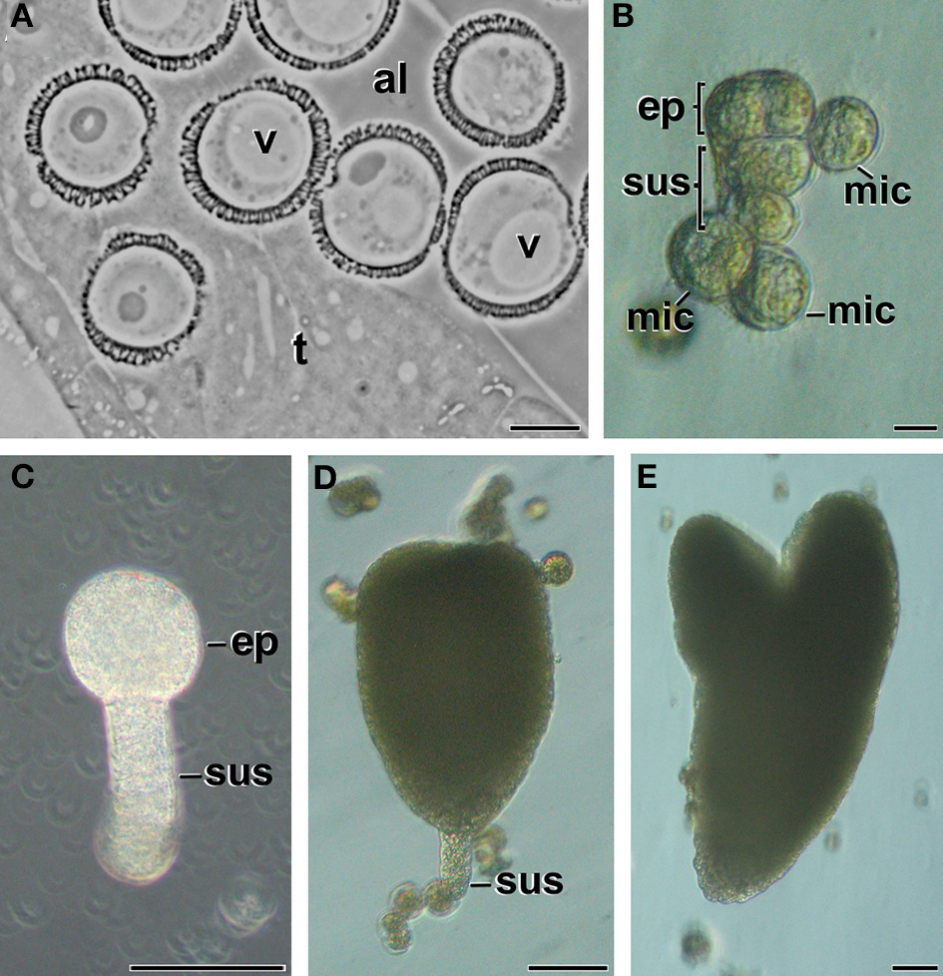
**Stages of *B. napus* microspore embryogenesis**. **(A)** Vacuolate microspores during *in vivo* development within the anther. **(B)** Just induced, embryogenic microspore showing two clearly differentiated domains, the embryo proper domain (ep) and the suspensor (sus). Other microspores (mic) are not sensitive to induction and become arrested or enter a pollen-like development. **(C)** Globular MDE. **(D)** transitional MDE. **(E)** Torpedo MDE. al, anther locule; t, tapetum; v, vacuole. Bars: **(A,B)**: 10 μm, **(C–E)**: 50 μm.

In this work, we performed *B. napus* isolated microspore cultures and analyzed the ultrastructure of DNA-containing organelles (plastids and mitochondria) during the different stages of the cultures, including before and after microspore isolation and induction. The first stage studied was the vacuolate microspore *in vivo*, still within the anther (Figure 1A). The identification and isolation of this particular developmental stage is essential for a successful induction, since it is known that this is the stage where the microspore is most sensitive to reprogramming treatments (Maraschin et al., 2005; Seguí-Simarro and Nuez, 2008; Dunwell, 2010). Just after the isolation, cultures exhibited mainly vacuolate microspores, as expected. Once induced, some microspores underwent multiple changes that transformed their morphology and architecture. As seen in Figure 1B, these microspores entered an embryogenic program defined by several cell divisions that generate embryo-like structures where the embryo proper and suspensor domains can be distinguished. In parallel, other microspores, not sensitive to induction, developed as pollen-like cells or just arrested in development (*mic* in Figure 1B). Dividing structures progressed as MDEs through the different stages of embryo development, including globular (Figure 1C), transitional (Figure 1D), heart-shaped and torpedo (Figure 1E) embryos, which showed an anatomy and external morphology remarkably similar to zygotic embryos (Seguí-Simarro and Nuez, 2008). Samples of all the stages shown in Figure 1 were collected at different culture stages, processed by HPF/FS, and observed under a TEM.

### Non-induced cells presented normal development of plastids

**Figure 2:**
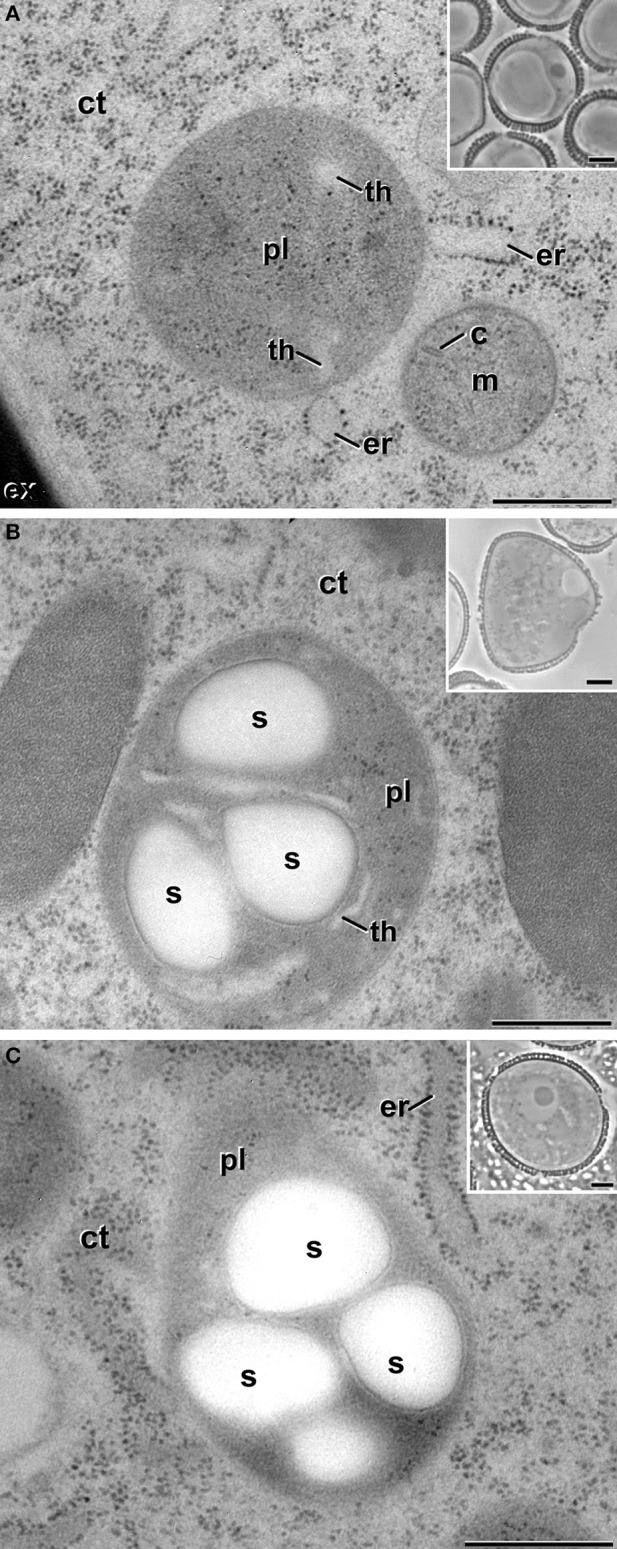
**Plastids (pl) of non-embryogenic *B. napus* cells**. **(A)** Proplastid of a vacuolate microspore during *in vivo* development within the anther. **(B)** Amyloplast of an *in vitro* cultured, pollen-like structure. **(C)** Amyloplast of a pollen grain within the anther. Insets show light microscopy sections of the corresponding stages. **(C)**, mitochondrial crista; ct, cytoplasm; er, endoplasmic reticulum; ex, exine; m, mitochondria; s, starch; th, thylakoid. Bars: 500 nm, insets: 5 μm.

Vacuolate microspores, still within the anther or just after isolation (not yet induced; Figure 2A), presented only proplastids, still undifferentiated. Proplastids were typically round. Their stroma appeared less electron dense than in the rest of stages studied, and presented very few thylakoids. Pollen-like structures presented plastids clearly transformed into amyloplasts, as revealed by the large starch deposits present in most of the plastids (Figure 2B). Amyloplasts of pollen-like cells presented different sizes and shapes, rounded, and/or elongated, and accumulated one or more starch granules. These amyloplasts were remarkably similar to those found in *in vivo* pollen grains within the anther (Figure 2C). In summary, non-induced cells presented conventional proplastids and amyloplasts, similar to those previously described for equivalent stages in this and other species (Sangwan and Sangwan-Norreel, 1987; Zaki and Dickinson, 1990; Satpute et al., 2005).

### Embryogenic microspores presented atypical plastids

**Figure 3:**
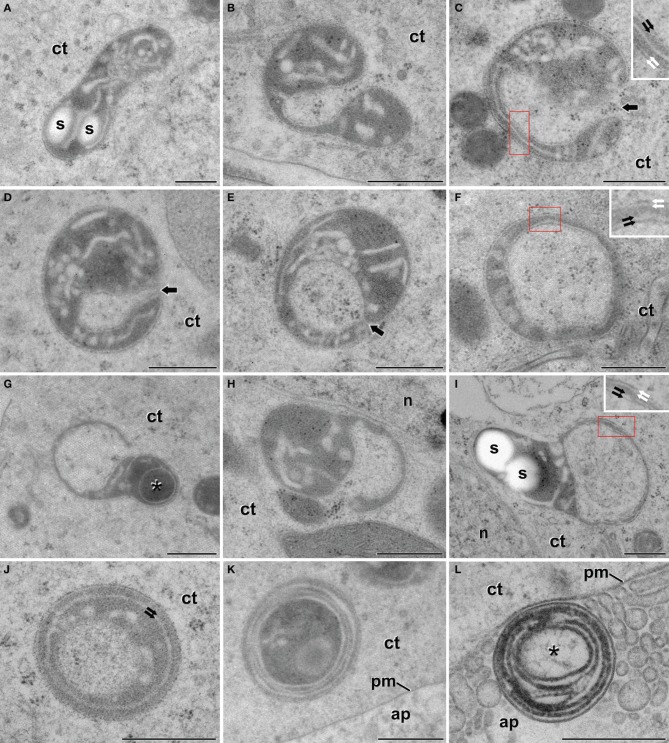
**Plastids of *B. napus* embryogenic microspores**. **(A)** Shows a conventional, elongated plastid, with two small starch deposits (s). **(B)** Dumbbell-shaped plastid curled to engulf a small cytoplasm region. **(C,D)** Plastids wrapped around a cytoplasmic region. Arrows indicate the thin cytoplasmic channel that still connects the engulfed cytoplasm. **(E)** Plastid engulfing a cytoplasmic region. Note that the connection between the trapped region and the cell cytoplasm is almost broken, but the point of closure is still evident (arrow). **(F)** Plastid showing an isolated cytoplasmic region. **(G,H)** Plastids engulfing large cytoplasmic regions. The asterisk in **(G)** indicates an electron dense cytoplasmic compartment. **(I)** Deformed plastid with a large cytoplasmic region entirely engulfed. **(J)** Plastid where the internal compartment contains fibrillar material, different from the surrounding cytoplasm. Note the presence of two concentric membranes below the plastid envelope (arrows). **(K)** Plastid showing numerous concentric membranes and a dark and disorganized contents, both in and out of the internal compartment. Note the proximity to the apoplast (ap). **(L)** Multilamellar body at the apoplast (ap), surrounded by excreted cellular debris. The asterisk indicates a internal compartment with traces of fibrillar material. The red boxes in **(C,F,I)** correspond to the area enlarged in the corresponding inset, where a double membrane system can be seen at both the outern plastid envelope (white arrows) and the inner envelope of the engulfed cytoplasm (black arrows). ct, cytoplasm; n, nucleus; pm, plasma membrane. Bars: 500 nm.

After the inductive period, some microspores underwent multiple changes that transformed their morphology and ultrastructure. As seen in Figure 1B, these microspores entered an embryogenic program defined by several cell divisions that generated embryo-like structures. A qualitative (Figure 3) and quantitative (Table 1) study of plastids of these cells revealed that 60.7% of them presented conventional shapes, including round, elongated, bean-like, or sausage-like profiles (Figure 3A). In all of these plastids, the stroma appeared more electron dense than in vacuolate microspores. In parallel, electron light tubular and/or cisternal profiles appeared loosely arranged within the stromal matrix, indicating the onset of thylakoid formation. Only in very few examples, small starch granules could be observed in these plastids (s in Figure 3A).

**Table 1:** **Quantitative analysis of plastids of embryogenic microspores**.

	**Number**	**Percentage (from total)**	**Percentage (from atypical)**
Conventional	142	60.7%	
Atypical	92	39.3%	
Engulfing (open profiles)	14	6.0%	15.2%
Engulfed (closed profiles)	63	26.9%	68.5%
Concentric	15	6.4%	16.3%
membranes/disorganized			
contents/multilamellar			
Total	234	100%	100%

However, many other plastids (39.3%) exhibited morphologies remarkably different from conventional (Figures 3B–I). The differences pertained principally to plastid shape and contents. As for plastid shape, the most striking difference was the presence of open plastid profiles surrounding cytoplasmic portions. Based on this observation, we identified two types of open plastid profiles. In the first type, we included elongated and curled dumbbell-shaped plastids (Figures 3B,C). Their extreme bending trapped portions of cytoplasm, as revealed by the presence of ribosomes and vesicles embedded in a matrix identical to that of the outer cytoplasm (Figure 3B). In the trapped cytoplasm, we never found cytoplasmic organelles such as plastids, mitochondria, Golgi stacks, or ER cisternae. In some cytoplasm-containing plastids, the connection between both cytoplasms was reduced to a thin channel or a pore of few nanometers (arrows in Figures 3C,E). In other plastids the channel was absent, suggesting that the membranes of opposite ends of the plastids were fused (Figure 3F), leaving a cytoplasmic portion isolated at the center of the closed plastid profile. The envelope of the internal cytoplasm-containing compartment was formed by a double membrane system identical to that found at the outer plastid envelope (insets in Figures 3C,F,I). Together, these observations suggested a dynamic process of plastid curling to engulf small regions of cytoplasm. The second type of profiles consisted of a relatively round plastid body engaged in the process of engulfing larger cytoplasmic areas (Figures 3G,H), or having them entirely engulfed (Figure 3I). In these cases, the large cytoplasmic area appeared in a lateral position within the deformed plastid, suggesting that the engulfment of large cytoplasmic areas imposes dramatic changes to the otherwise typical structure of the plastid. Overall, open plastid profiles suggesting engulfment of cytoplasm accounted for 15.2% of the atypical plastid profiles we identified.

Among all the atypical plastid profiles, the most frequent (68.5%) were those containing isolated cytoplasm portions. Most of these closed plastid profiles showed no structural abnormalities other than the engulfed cytoplasm. However, some of them presented more electron dense contents, indicating the onset of a change in the engulfed cytoplasm. Occasionally, plastids with an electron dense content (asterisk in Figure 3G) were engaged in a second round of cytoplasmic engulfment, suggesting that this might be a recurrent process. In addition, others showed one or more concentric membranous structures surrounding the cytoplasmic compartment (Figure 3J). Most of the plastids with several concentric membranes presented dark and disorganized contents as well (Figure 3K). Interestingly, multilamellar bodies of a size similar to the plastids with concentric membranes were also found in the cytoplasm, close to the plasma membrane (Figure 3K), and in the apoplast (Figure 3L), together with cellular debris excreted as a consequence of embryogenesis induction (Corral-Martínez et al., 2013). These multilamellar bodies presented an internal compartment with fibrillar material, similar to that present in lytic compartments. Closed plastid profiles with concentric membranes, dark, fibrillar, and disorganized contents, together with cytoplasmic and apoplastic multilamellar bodies, accounted for 16.3% of the atypical profiles observed. Altogether, these plastid profiles suggested the occurrence of plastid degradation and excretion out of the cell.

### 3-D reconstruction of subcellular volumes of embryogenic microspores

**Figure 4:**
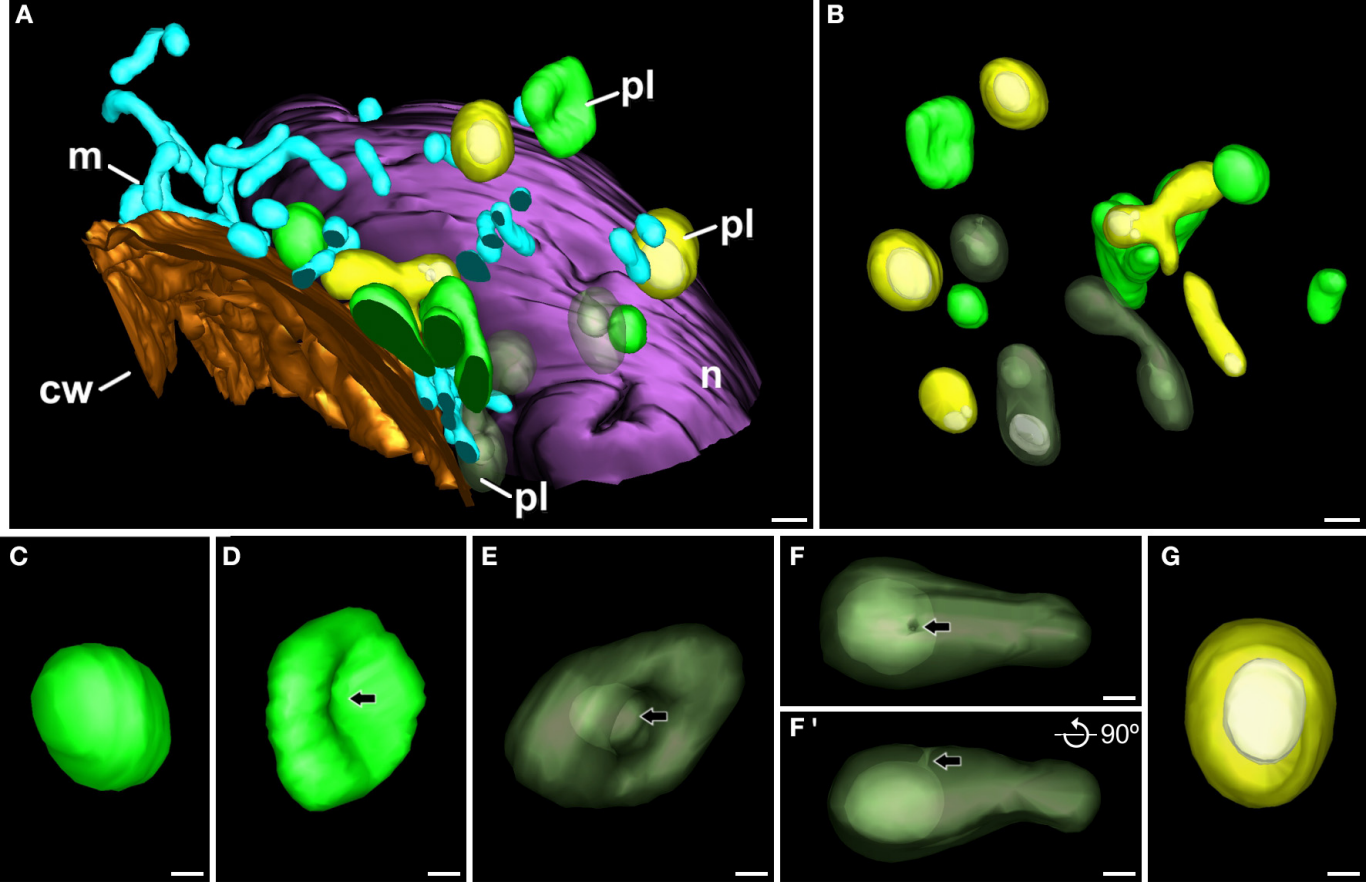
**3-D model of a subcellular volume of a *B. napus* embryogenic microspore**. **(A)** Modeled subcellular volume. **(B)** Model excluding all the cell structures but the plastids (pl). The different plastid types are modeled in different colors: conventional (light green), open profiles engulfing cytoplasm (dark green), and closed profiles (yellow) with the engulfed cytoplasm (white). **(C)** Conventional, round-shaped plastid. **(D)** Disc-shaped plastid with a slight central depression (arrow). **(E)** Plastid starting to engulf cytoplasm. The arrow points to a deep depression that creates a cytoplasmic pocket within the plastid. **(F,F')** Cytoplasm-containing plastid where the internal cytoplasm is connected with the outer cytoplasm just by a narrow channel that ends in a small pore at the plastid surface. **(F')** is a 90° turn of this plastid, for a clear visualization of the narrow channel. Arrows point to the pore in **(F)** and to the narrow channel in **(F')**. **(G)** Round plastid (yellow) with the cytoplasmic contents (white) entirely isolated from the outer cytoplasm. cw, cell wall; m, mitochondrion; n, nucleus. Bars: **(A,B)**: 500 nm; **(C–G)**: 200 nm.

Theoretically, it might be possible that the atypical plastid profiles observed in TEM micrographs of embryogenic microspores correspond to polar sections of cup-shaped plastids. Alternatively, these plastid profiles might correspond to equatorial sections of ring-shaped plastids. In other words, the atypical plastid profiles we observed might be artifactual, and might not engulf cytoplasm actually. In order to rule out this possibility, and to figure out the actual 3-D structure of these plastids, we performed FESEM-FIB-based 3-D reconstructions and models of large cytoplasmic areas of embryogenic microspores (Figure 4A; Supplementary Movie S1). These models confirmed the presence of three morphologically different plastid types (Figure 4B), as previously observed in TEM micrographs. We modeled each plastid type in different colors. Plastids with conventional morphologies (oval, round, or elongated, not engulfing cytoplasm) were modeled in light green (Figures 4B–D). Some of them were round or oval (Figure 4C), and others exhibited a disc-like morphology with a slight central depression (arrow in Figure 4D; yellow arrow in Supplementary Movie S2), suggesting the onset of a process of membrane invagination. Plastids engulfing cytoplasm were modeled in dark green (Figures 4B,E–F'). These plastids presented different sizes and shapes. Some of them, similar in shape to the disc-shaped conventional plastid of Figure 4D, showed a profound invagination of their envelope, generating a cytoplasm-containing open pocket (arrow in Figure 4E, central plastid in Supplementary Movie S2). Other plastids were oval or elongated, and engulfed large portions of cytoplasm. In these plastids, the cytoplasm outside and inside the plastid was connected by a narrow channel of a few nanometers, leaving a pore at the surface of the plastid (arrows in Figures 4F,F'; Supplementary Movie S3 and red arrow in Supplementary Movie S2). The third plastid type, modeled in yellow (Figures 4B,G) showed an oval or elongated shape, and included one or more cytoplasm portions (modeled in white) fully isolated from the outer cytoplasm, as evidenced by the absence of connecting channels (Figure 4G, Supplementary Movies S4, S5). Together, the different plastids observed in 3-D models (Figures 4C–G) confirmed our observations from 2-D TEM micrographs, and indicated the occurrence of a mechanism whereby some plastids surround and engulf discrete cytoplasm regions, isolating them from the outer cytoplasm.

We also observed that cytoplasm engulfment was associated to a change in plastid morphology. In conventional plastids and in atypical plastids with open profiles (inner and outer cytoplasm still connected), elongated morphologies were more frequent than round morphologies, accounting for 57% of the conventional plastids and 71% of atypical plastids with open profiles. In the second case, the cytoplasmic volume was considerably smaller than the total plastid volume. In contrast, most (77%) of the atypical plastids with closed profiles (internal cytoplasm entirely isolated from the outer cytoplasm), showed round morphologies, and their cytoplasmic content appeared to occupy a larger fraction of the plastid volume (Figure 4G). These observations suggested that cytoplasm engulfment induced a change not only in plastid shape, but also in internal architecture, reducing the stromal volume compared to that of the engulfed cytoplasm.

**Figure 5:**
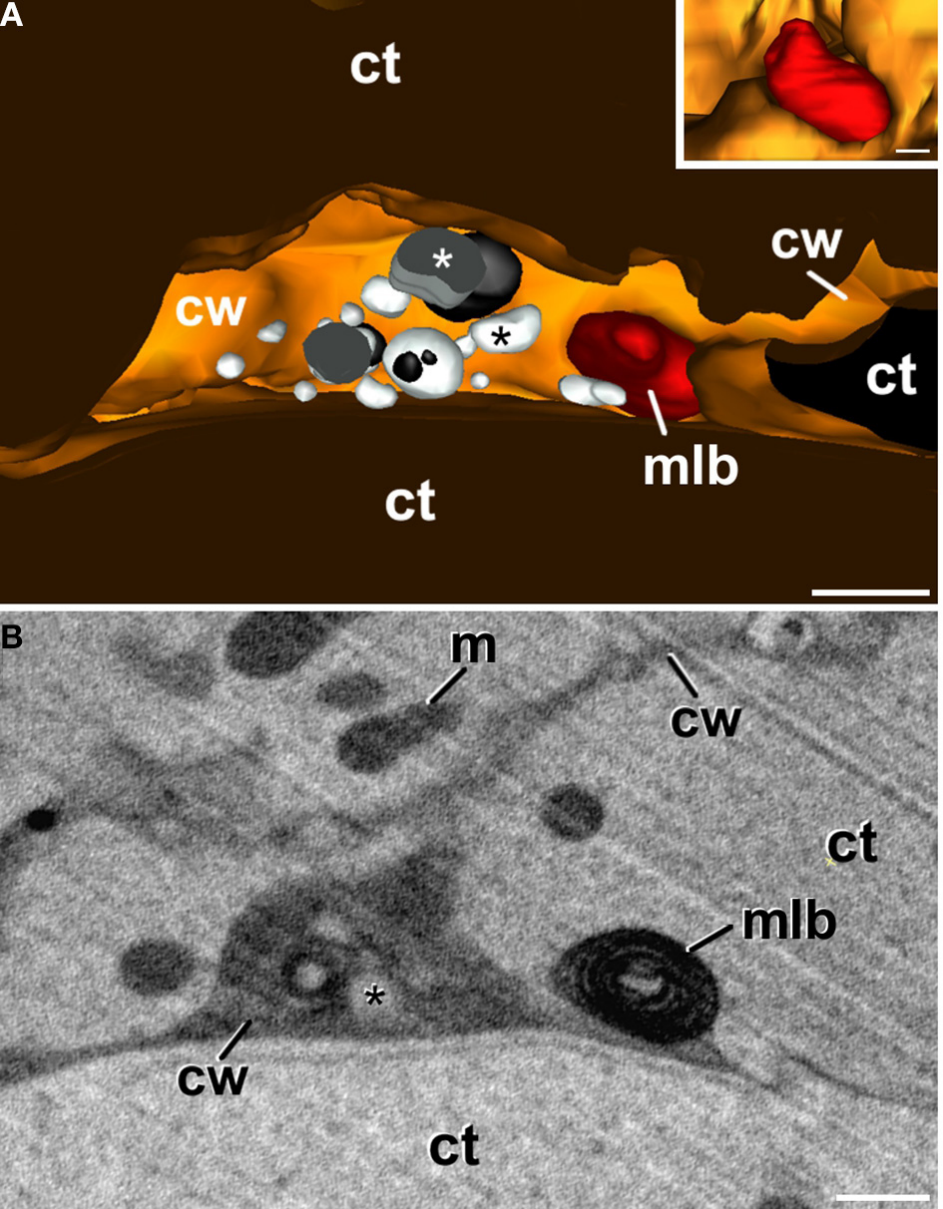
**3-D reconstruction of a region from a newly-formed cell wall of a *B. napus* embryogenic microspore**. **(A)** is a 3-D model showing the apoplast with several vesicular bodies of different electron density (white and black asterisks), and an excreted plastid, degraded to become a multilamellar body (mlb). The inset shows the plastid from a different perspective. Note that part of the plastid is tightly surrounded by the plasma membrane whereas the other part is facing the apoplast. **(B)** One of the FESEM-FIB micrographs used for the 3-D reconstruction showing the multilamellar body separated from the excreted cytoplasmic vesicular bodies (asterisk), embedded in a matrix of dense material. ct, cytoplasm; cw, cell wall; m, mitochondrion. Bars: 500 nm.

In addition to conventional and atypical plastids, we also modeled the multilamellar bodies excreted to the apoplast (Figure 5). Figure 5A shows a model of an apoplast region and Figure 5B shows one of the several FESEM-FIB micrographs used to reconstruct it. This region corresponds to a swollen apoplast area with numerous membranous bodies embedded in a matrix of dense material (Corral-Martínez et al., 2013). Close to this area but separate from it, a multilamellar body (inset in Figure 5A, modeled in red; Supplementary Movie S6) similar to those observed in TEM images (Figures 3L, 5B) is observed. The multilamellar body is similar in size and shape to the atypical plastids and multilamellar bodies observed in the cytoplasm. The 3-D model showed that nearly half of the body is facing the apoplast, while the other half is still tightly wrapped by the plasma membrane, which clearly delineates the shape of the body (inset in Figure 5A). These observations are suggestive of a process of excretion of the multilamellar body, likely mediated by the fusion of its outer membrane with the plasma membrane, and independent of the excretion of the membranous and dense material.

### Cytoplasm-containing plastids showed acid phosphatase activity

**Figure 6:**
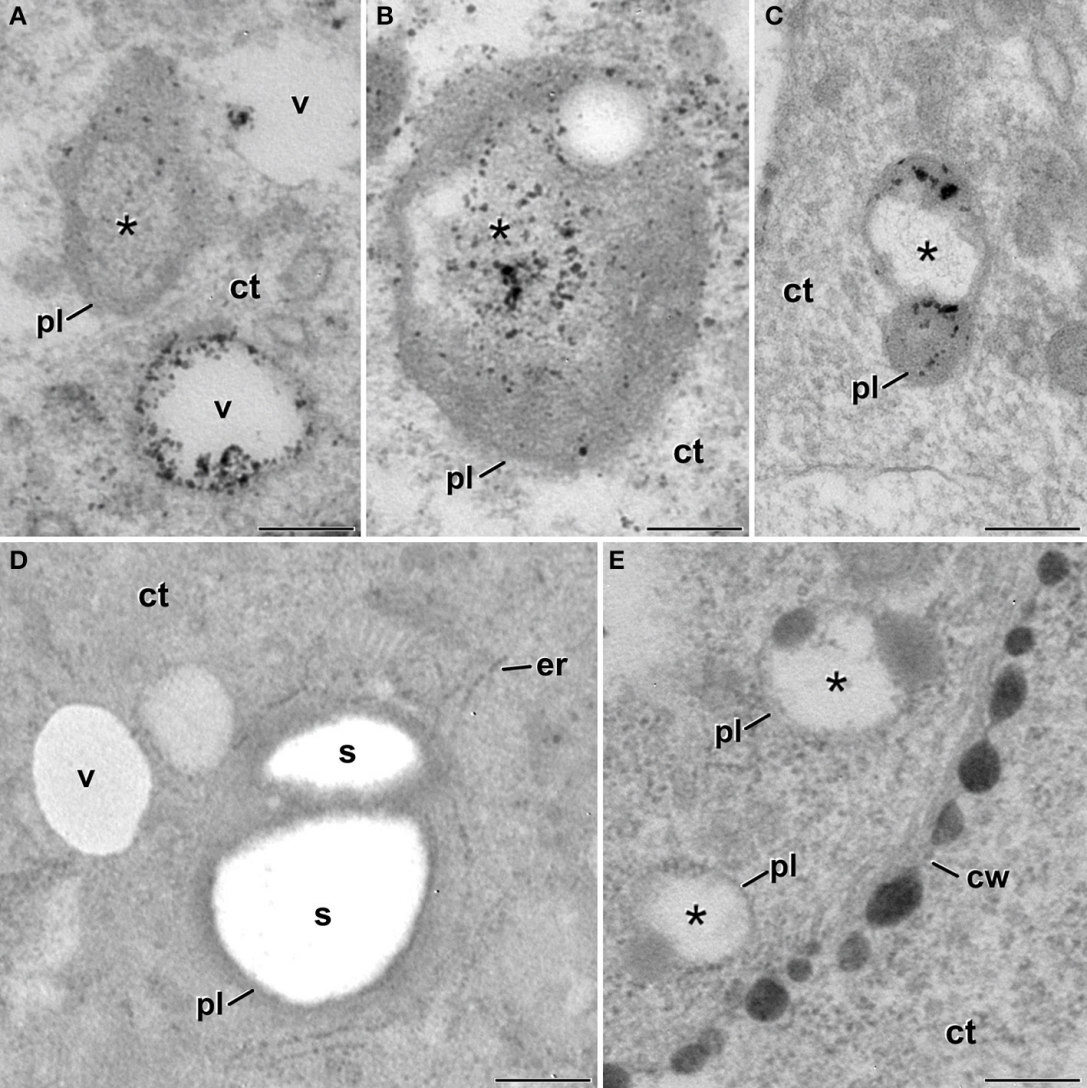
**Detection of acid phosphatase activity in cytoplasm-containing plastids of *B. napus* embryogenic microspores**. **(A)** Cytoplasm-containing plastid (pl) showing a discrete acid phosphatase activity as revealed by the small and scarce dense precipitates. The cytoplasm compartment (asterisk) is devoid of precipitates. As a reference, two adjacent vacuoles (v) show different amounts of precipitates. **(B)** Cytoplasm-containing plastid where most of the dense precipitates concentrate within the cytoplasm compartment (asterisk). **(C)** Plastid where all the cytoplasmic contents seems digested, and the dense precipitates (lytic activity) concentrate over the plastidial stroma. **(D)** Vacuoles and amyloplasts of a non-embryogenic, pollen-like structure. Note the total absence of dense precipitates. **(E)** Cytoplasm-containing plastids of a control sample without β-glycerophosphate. Note the total absence of dense precipitates. ct, cytoplasm; cw, cell wall; er, endoplasmic reticulum; s, starch. Bars: 250 nm.

As seen in Figures 3G,J–L, some of the cytoplasm-containing plastids showed signs of degradation of their cytoplasmic contents and of the entire plastid. In order to elucidate a putative lytic activity in these organelles, similar to that found in autophagosomes of the cytoplasm of embryogenic microspores (Corral-Martínez et al., 2013), we performed an *in situ* acid phosphatase cytochemical assay (Figure 6). In embryogenic microspores we observed cytoplasm-containing plastids with different amounts of electron dense precipitates, indicative of different levels of acid phosphatase activity. Figure 6A shows a plastid containing cytoplasm similar to that found out the plastid, together with few small precipitates distributed throughout the plastid, indicating a mild lytic activity. As a reference, this figure also includes a lytic cytoplasmic vacuole with an electron light lumen and numerous precipitates, indicating a more intense lytic activity. Figure 6B shows a plastid where most of the precipitates concentrate in the engulfed cytoplasm, suggesting that lytic activity is first initiated in the cytoplasmic cargo. Figure 6C shows a plastid where the cytoplasmic content seems already digested, as revealed by the electron translucent internal compartment, similar to that of lytic vacuoles (Figure 6A). This plastid exhibits electron dense precipitates dispersed throughout the stroma. Together, Figures 6A–C are suggestive of a process whereby cytoplasm-containing plastids first digest their cytoplasm, and then enter an auto-lytic process conducing to the entire degradation of the plastid. In contrast, pollen-like structures present in the same sections and therefore exposed to the same cytochemical assay did not show any precipitate, neither in their amyloplasts nor in their cytoplasmic vacuoles (Figure 6D). Controls excluding β-glycerophosphate did not show any comparable precipitate in any of the studied cell types (Figure 6E).

### MDEs presented conventional plastids

**Figure 7:**
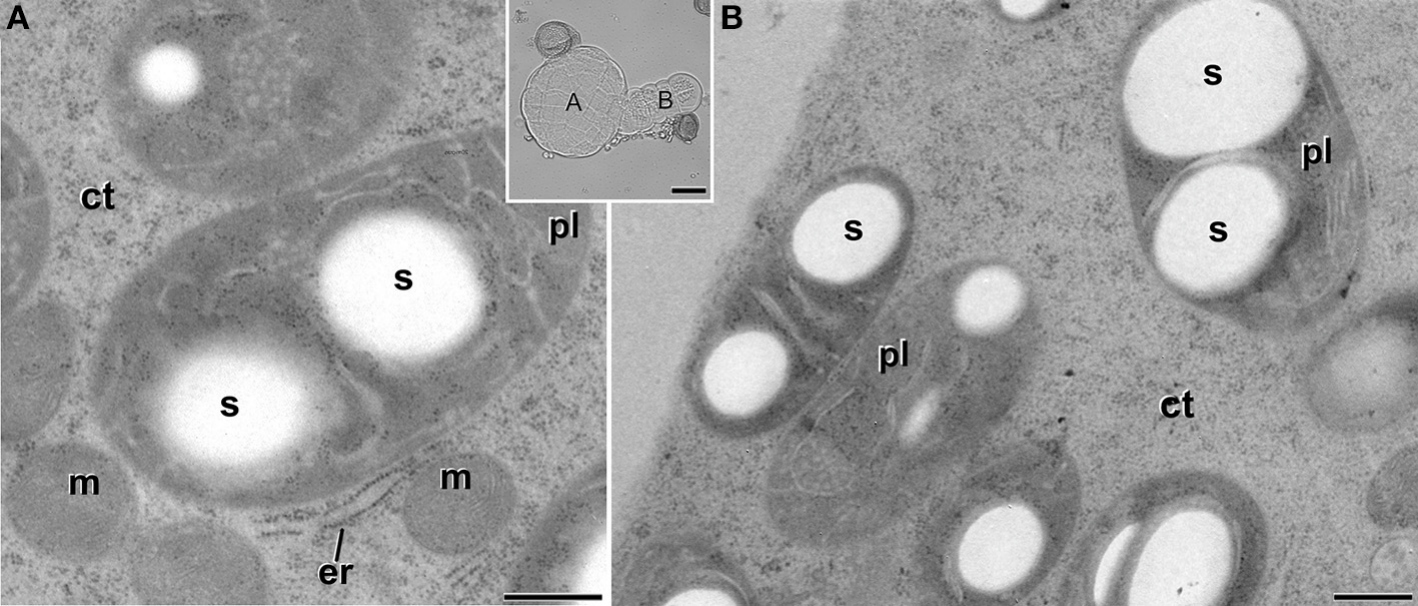
**Starch-containing plastids (pl) from *B. napus* globular MDEs**. The inset shows a light microscopy section of a globular MDE where the embryo proper **(A)** and the suspensor **(B)** domains can be clearly differentiated. Panels **(A,B)** show electron micrographs of plastids of embryo proper **(A)** and suspensor **(B)** cells. ct, cytoplasm; er, endoplasmic reticulum; m, mitochondria; s, starch. Bars: **(A,B)**: 500 nm. Inset: 20 μm.

Embryogenic structures progressed as MDEs through the different stages of embryo development, including globular (Figure 1C), transitional (Figure 1D), heart-shaped, and torpedo (Figure 1E) MDEs. Plastids from cells of these MDE stages were also analyzed in order to check whether the unusual plastid profiles found in induced microspores persisted during further MDE development, or they were transient structures, exclusive of the first stages of MDE induction. As seen in Figure 7, the plastids found in the embryo proper domain (Figure 7A) and in the suspensor (Figure 7B) of globular MDEs were similar to those found in pollen grains (Figure 2C). All of them exhibited a round or oval shape, dense stroma, tubular, and/or lamellar thylakoids, and starch granules. No engulfed cytoplasmic regions were observed in any case. Cells of heart-shaped, transitional, and torpedo MDEs (data not shown) presented only conventional starch and thylakoid-containing plastids, structurally equivalent to those described for globular MDEs. Thus, it seemed that the unusual features observed in plastids of embryogenic microspores did not persist during MDE development. Instead, plastids adopted a conventional architecture, characterized by the presence of starch and thylakoids.

### Mitochondria present a conventional architecture during all the stages of microspore embryogenesis

**Figure 8:**
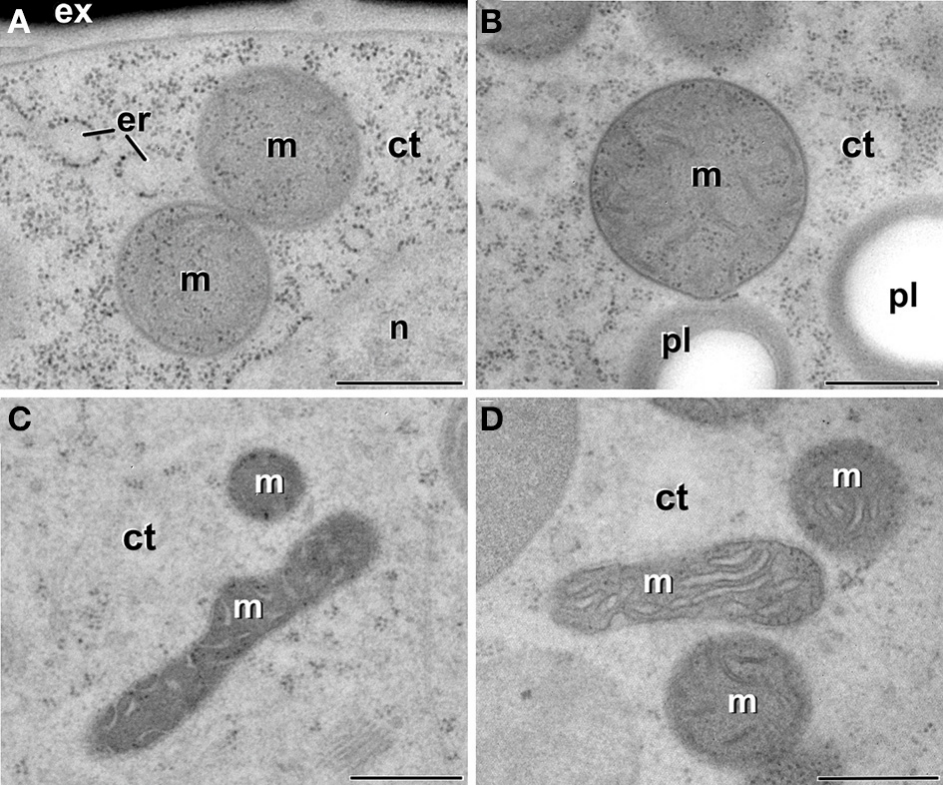
**Mitochondria (m) of *B. napus* vacuolate microspores during *in vivo* development within the anther (A), pollen grains within the anther (B), embryogenic microspores (C), and globular MDEs (D)**. ct, cytoplasm; er, endoplasmic reticulum; ex, exine; pl, plastid; n, nucleus. Bars: 500 nm.

In order to check whether the atypical features found in plastids of embryogenic microspores were extensive to other subcellular organelles, we also analyzed the ultrastructure of mitochondria in all the culture stages processed. Mitochondria of vacuolated microspores and pollen grains within the anther (Figures 8A,B) presented a conventional oval or sausage-like morphology, and a mitochondrial matrix where cristae can be easily identified as emerging from the inner mitochondrial membrane. This description also applied to microspores of pollen-like structures developing *in vitro* (data not shown), as well as of just induced, embryogenic microspores (Figure 8C) and MDEs (Figure 8D). Therefore, in contrast to the changes observed in plastids of embryogenic microspores, the mitochondria of embryogenic microspores presented a morphology and architecture equivalent to those of cells of any other culture stage, as well as of non-cultured *B. napus* cells.

## Discussion

### The androgenic switch produces atypical plastids

We showed in this work that induction of embryogenesis produces dramatic changes in proplastids of embryogenic microspores, diverting them from their original fate (pollen amyloplasts), and transforming them into different, unique structures. It could be argued that this change in plastid architecture and function could be a side consequence of the stress treatment applied and the *in vitro* culture environment, since it is known that *in vitro* culture may alter the normal structure and function of plant cells. However, it must be noted that *in vitro* microspore cultures produce not only embryogenic microspores, but also pollen-like structures, also submitted to same stressing conditions, but containing only amyloplasts, and not cytoplasm-containing plastids. In addition, *in vitro* developed MDEs showed only conventional plastids, indicating that the plastidial changes are a transient phenomenon. Furthermore, other DNA-content organelles such as mitochondria, present in the same cells that developed atypical plastids, did not undergo any change in response to the *in vitro* culture. Therefore, it seems clear that the occurrence of cytoplasm-containing plastids is not related to the *in vitro* culture conditions, including the heat shock treatment used to induce microspore embryogenesis. Instead, we postulate that the occurrence of this unusual plastid type is inherent to the androgenic switch. The biological significance and role in the context of microspore embryogenesis is discussed next.

### Cytoplasm-containing plastids of embryogenic microspores are engaged in autophagy

A plastidial architecture similar to that described hereby for *B. napus* embryogenic microspores has been rarely reported in the literature. Similar observations have only been reported in plastids of suspensor cells of *P. coccineus* (Nagl, 1977) and *P. vulgaris* (Gärtner and Nagl, 1980), where plastids transformed into autophagic vacuoles during the senescence of the suspensor, and in petal cells of *Dendrobium*, where it was shown that plastids adopt autophagic functions, engulfing, and digesting portions of the cytoplasm (van Doorn et al., 2011). As seen, all these reports established a clear link between these plastid transformations and their engagement in autophagy. Our TEM images and 3-D reconstructions demonstrated that a significant percentage of the plastids of embryogenic microspores engulf and isolate entire organelle-free cytoplasmic portions, creating an independent intraplastidial compartment. Structural changes such as the reduction in the stromal volume and the number of thylakoids, suggests the onset of a new role for the cytoplasm-containing plastids. As seen in Figure 3, the compartmentalized cytoplasm is surrounded by a double membrane system structurally identical to the plastid envelope. This suggests that the intraplastidial compartment is originated from the plastidial double membrane. The 3-D models confirmed the physical continuity between the plastid envelope and the cytoplasmic pockets or internal cytoplasmic compartments. Such double membrane-bound compartments are remarkably similar to the widely described plant autophagosomes (Aubert et al., 1996; Otegui et al., 2005; Lundgren Rose et al., 2006; Reyes et al., 2011). The abundance of C-shaped and dumbbell-shaped plastid profiles found in embryogenic cells, together with the 3-D plastid models, suggests that the intraplastidial compartment is formed by curling and protrusion of a disc-shaped plastid, which wraps around a cytoplasm portion and eventually fuse their opposite ends to engulf it. Mechanistically, this process is also resembling the process of autophagosome formation in plant cells (Li and Vierstra, 2012), and specifically in *B. napus* embryogenic microspores, as a consequence of embryogenesis induction (Corral-Martínez et al., 2013). Furthermore, the acid phosphatase activity demonstrated not only in the internalized cytoplasm but also in the stroma, confirmed the lytic activity of these organelles. Based on all these evidences, it is reasonable to assume that we are observing plastids acting as autophagosomes and developing internal autophagic compartments that eventually lead to the digestion of the entire plastid. According to the term coined by Nagl (1977), they would be *plastolysomes*.

### Plastolysomes of embryogenic microspores are excreted to the apoplast

**Figure 9:**
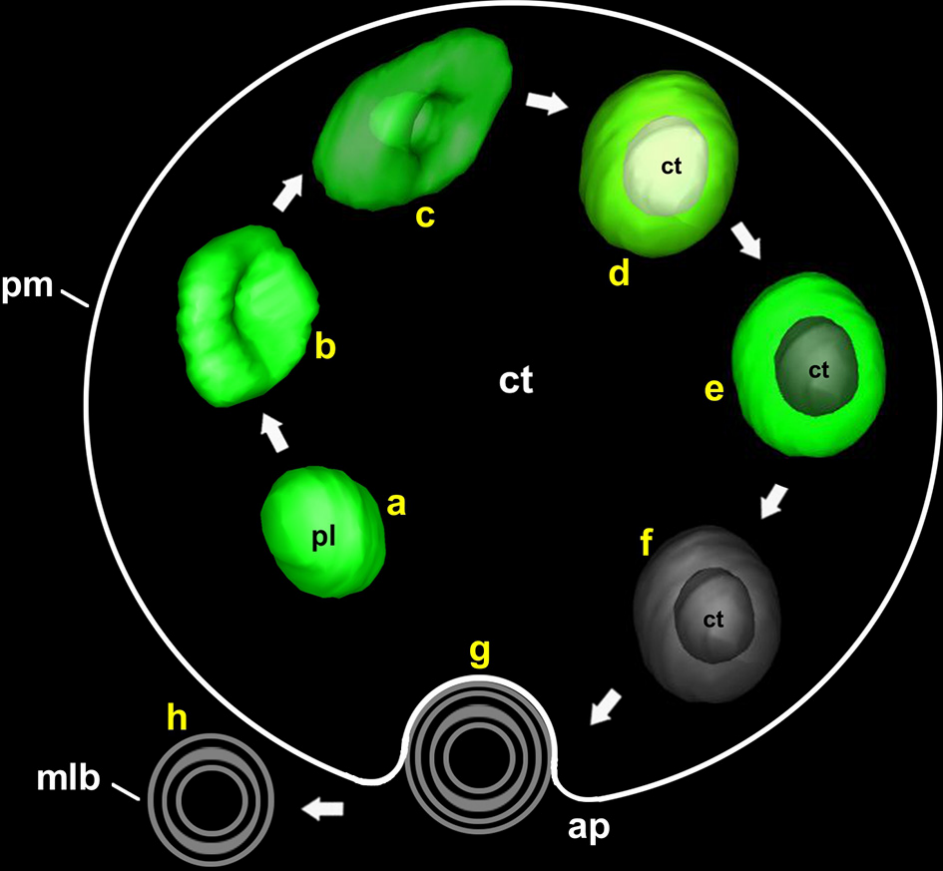
**Model of the different steps involved in the process of plastolysome formation and excretion to the apoplast**. First, an initially conventional plastid (a) creates an invagination at its envelope (b). The invagination enters into the plastid until it wraps the cytoplasmic portion (c). Then, the plastid envelope closes around the cytoplasmic portion, isolating it from the outer cytoplasm (d). Once the cytoplasm is internalized, digestion of the cytoplasm (e) and then of the entire plastid (f) takes place. Finally, the remnants are excreted to the apoplast (g,h) in the form of a multilamellar body. ap, apoplast; ct, cytoplasm; mlb, multilamellar body; pl, plastid; pm, plasma membrane.

In *Dendrobium* and *Phaseolus*, the lytic plastidial compartments were filled with material of different levels of electron density, from dark to very light (Nagl, 1977; Gärtner and Nagl, 1980; van Doorn et al., 2011). This, together with the demonstration of acid phosphatase activity, led the authors to propose that the engulfed cytoplasm was digested and recycled. In embryogenic microspores, despite that many of the atypical plastids presented few or no structural evidences of digestion, evidences of acid phosphatase activity was detected in many of them (Figures 6A,B). Furthermore, full plastid degradation, similar to those of *Dendrobium* and *Phaseolus* cells, was found in ~16% of the atypical plastids, and multilamellar bodies, similar in size to plastids, were found in the cytoplasm and also out of it, in the apoplast. Figure 5 illustrates one of these bodies being excreted to the apoplast in a way that indicates that the body has been excreted independently, and not as part of a massive excretion of cytoplasmic material. All these observations made us think that the fate of cytoplasm-containing plastids would not only be their degradation, but also their excretion out of the cell. We propose that cytoplasm engulfment affects not only the structure of the plastid, but also its fate and subsequently, its function. The cytoplasm engulfment, the lytic activity, and the loss of stroma and grana may be related to a functional shift of the plastid, transforming itself into a vehicle for cytoplasm and plastid elimination through an orchestrated sequence of steps that involves cytoplasm engulfment, cytoplasm and plastid digestion, and finally excretion of the entire structure (Figure 9).

Recently, similar processes of autophagy associated to excretion of the digested material were demonstrated to occur in embryogenic microspores of *B. napus* (Corral-Martínez et al., 2013). It was proposed that these combined processes would be acting as a cleaning mechanism for massive removal of useless cytoplasmic material. However, this study showed few evidences of the usefulness of this process to eliminate plastids. In other words, autophagosome formation and excretion would not account for the reduction in the number of plastids previously described as associated to embryogenesis induction (reviewed in Shariatpanahi et al., 2006; Makowska and Oleszczuk, 2014). Therefore, it seems reasonable to speculate that the processes described by Corral-Martínez et al. (2013) and hereby (formation and excretion of autophagosomes and plastolysomes) could be related as parallel parts of a *cleaning program* necessary to adapt the embryogenic microspore to its new developmental scenario (Seguí-Simarro and Nuez, 2008). In this context, plastolysome-mediated autophagy and excretion would be responsible for the elimination of small cytoplasm portions and, most importantly, ~40% of the plastids, according to our quantification of the plastids that enter this pathway. The cellular mechanisms, still unknown, that mediate the excretion of entire autophagosomes, might also govern the excretion of plastolysomes, even before their contents have been entirely digested and recycled. Further research should focus on elucidating such putative mechanisms and the molecular link between these two parallel ways of cytoplasmic cleaning.

### Is there a possible link between plastolysome formation and androgenic recalcitrance?

In general, angiosperm microspores have only proplastids, which transform into amyloplasts in pollen grains (Clément and Pacini, 2001). This general rule, however, has some exceptions. In 1987, Sangwan and Sangwan-Norreel studied the relationship between the androgenic response of several species and the plastid types present in their microspores and pollen grains. They observed that responsive species present proplastids up to the bicelullar pollen stage (the latter inducible stage). In contrast, in recalcitrant species proplastids differentiate to amyloplasts at the microspore stage or even before. Indeed, the differentiation of proplastids into amyloplasts has been related to the lack of embryogenic response of late pollen stages (Maraschin et al., 2005) and of recalcitrant, non-responsive species (Sangwan and Sangwan-Norreel, 1987). It is known that inhibition of starch biosynthesis blocks pollen development (Datta et al., 2002), whereas the presence of starch-free proplastids is needed for a microspore to be sensitive to induction (Nitsch and Nitsch, 1969; Sangwan and Sangwan-Norreel, 1987). Thus, it appears that starch biosynthesis in amyloplasts is a marker of irreversible microspore commitment toward gametogenesis (Clément and Pacini, 2001). In maize, a protein analysis during microspore development revealed that as soon as starch biosynthesis initiates in the plastids of microspores (not yet pollen), there is a drastic decrease in the synthesis of a number of polypeptides whereas other, new ones, are being synthesized *de novo* (Mandaron et al., 1990). These authors related this starch-mediated change in the pattern of protein synthesis with the androgenic competence.

Our results would add a new piece to this puzzle, explaining why amyloplasts-containing microspores cannot undergo embryogenesis: an amyloplast would not be able to transform into a plastolysome by the time when the stress treatment is applied. Therefore, amyloplast-containing cells would not be able to execute the cytoplasmic cleaning process as extensively as those having proplastids, not yet differentiated. Conversely, it could be speculated, according to the hypothesis of Mandaron et al. (1990), that the activation of the genes for starch biosynthesis might eliminate proteins necessary for embryogenesis induction and/or produce inhibitors of embryogenesis that make microspores lose their embryogenic potential. In this context, the process we hereby describe would greatly contribute to reduce the impact of starch biosynthesis in the blockage of embryogenesis by targeting plastids to elimination through their engagement in autophagy. Obviously, these all are speculations that should be confirmed in further studies. We are currently examining HPF/FS-processed embryogenic microspores from other species, including recalcitrant crops, in order to validate the link found hereby between plastid elimination and embryogenic competence, and to verify to what extent the formation of plastolysomes is a common feature of microspore embryogenesis.

## Author contributions

JMSS designed the research. VPV, ARS, and JMSS obtained and processed the samples. VPV and PCM performed the experiments. VPV and JMSS analyzed the data and wrote the manuscript.

### Conflict of interest statement

The authors declare that the research was conducted in the absence of any commercial or financial relationships that could be construed as a potential conflict of interest.

## Supplementary material

Supplementary Movie S1 **One of the models produced from 3-D reconstructions showing all of the subcellular structures present in the reconstructed volume**. The nucleus is modeled in purple, the newly formed cell plate in dark yellow, the exine in red, the mitochondria in light blue, the vacuoles in white, and the different plastid types in different colors: conventional in light green, open profiles engulfing cytoplasm in dark green and closed profiles in yellow with the engulfed cytoplasm in white.

Supplementary Movie S2 **A series of consecutive FESEM-FIB slices covering three entire plastids at different stages in the process of cytoplasm engulfment**. The disc-shaped plastid at the lower left quadrant is slightly bent, beginning to form a cytoplasmic pocket (yellow arrow) at one side. The central, large and elongated plastid is forming a deep cytoplasmic pocket around the cytoplasm being engulfed. The round plastid at the upper right quadrant engulfs a cytoplasm portion almost entirely, leaving a small pore (red arrow) that connects the inner and outer cytoplasm.

Supplementary Movie S3 **3-D model of cytoplasm-containing plastids where the cytoplasm outside and inside the plastid is not totally isolated, but still connected by narrow channels, leaving a pore at the surface of the plastids**.

Supplementary Movie S4 **3-D model of cytoplasm-containing plastids where the engulfed cytoplasm is entirely isolated from the outer cytoplasm**.

Supplementary Movie S5 **A series of consecutive FESEM-FIB slices covering an entire plastid containing a cytoplasmic portion totally isolated from the outer cytoplasm**.

Supplementary Movie S6 **A series of consecutive FESEM-FIB slices covering a multilamellar body (encircled by a red line) being excreted to the apoplast**. Note that approximately half of the body is still tightly wrapped by the plasma membrane, and the other half is exposed to the apoplast.

## References

[B1] Aubert S. Gout E. Bligny R. Marty-Mazars D. Barrieu F. Alabouvette J.. (1996). Ultrastructural and biochemical characterization of autophagy in higher plant cells subjected to carbon deprivation: control by the supply of mitochondria with respiratory substrates. J. Cell Biol. 133, 1251–1263. 10.1083/jcb.133.6.1251 8682862 PMC2120909

[B2] Clément C. Pacini E. (2001). Anther plastids in angiosperms. Bot. Rev. 67, 54–73 10.1007/BF02857849

[B3] Corral-Martínez P. Parra-Vega V. Seguí-Simarro J. M. (2013). Novel features of *Brassica napus* embryogenic microspores revealed by high pressure freezing and freeze substitution: evidence for massive autophagy and excretion-based cytoplasmic cleaning. J. Exp. Bot. 64, 3061–3075. 10.1093/jxb/ert151 23761486

[B4] Datta R. Chamusco K. C. Chourey P. S. (2002). Starch biosynthesis during pollen maturation is associated with altered patterns of gene expression in maize. Plant Physiol. 130, 1645–1656. 10.1104/pp.006908 12481048 PMC166680

[B5] Dunwell J. M. (2010). Haploids in flowering plants: origins and exploitation. Plant Biotechnol. J. 8, 377–424. 10.1111/j.1467-7652.2009.00498.x 20233334

[B6] Dunwell J. M. Sunderland N. (1974a). Pollen ultrastructure in anther cultures of *Nicotiana tabacum* I. Early stages of culture. J. Exp. Bot. 25, 352–361 10.1093/jxb/25.2.352

[B7] Dunwell J. M. Sunderland N. (1974b). Pollen ultrastructure in anther cultures of *Nicotiana tabacum* II. Changes associated with embryogenesis. J. Exp. Bot. 25, 363–373 10.1093/jxb/25.2.363

[B8] Dunwell J. M. Sunderland N. (1975). Pollen ultrastructure in anther cultures of *Nicotiana tabacum* III. The first sporophytic divisions. J. Exp. Bot. 26, 240–252 10.1093/jxb/26.2.240

[B9] Dunwell J. M. Sunderland N. (1976a). Pollen ultrastructure in anther cultures of *Datura innoxia*. I. Division of the presumptive vegetative cell. J. Cell Sci. 22, 469–480. 1018041 10.1242/jcs.22.3.469

[B10] Dunwell J. M. Sunderland N. (1976b). Pollen ultrastructure in anther cultures of *Datura innoxia*. II. The generative cell wall. J. Cell Sci. 22, 481–491. 1018042 10.1242/jcs.22.3.481

[B11] Dunwell J. M. Sunderland N. (1976c). Pollen ultrastructure in anther cultures of *Datura innoxia*. III. Incomplete microspore division. J. Cell Sci. 22, 493–501. 1018043 10.1242/jcs.22.3.493

[B12] Forster B. P. Heberle-Bors E. Kasha K. J. Touraev A. (2007). The resurgence of haploids in higher plants. Trends Plant Sci. 12, 368–375. 10.1016/j.tplants.2007.06.007 17629539

[B13] Gärtner P. J. Nagl W. (1980). Acid phosphatase activity in plastids (plastolysomes) of senescing embryo-suspensor cells. Planta 149, 341–349. 10.1007/BF00571168 24306370

[B14] Gilkey J. C. Staehelin L. A. (1986). Advances in ultrarapid freezing for the preservation of cellular ultrastructure. J. Electron Microsc. Tech. 3, 177–210 10.1002/jemt.1060030206

[B15] Hause B. Hause G. Pechan P. Van Lammeren A. A. M. (1993). Cytoskeletal changes and induction of embryogenesis in microspore and pollen cultures of *Brassica napus* L. Cell Biol. Int. 17, 153–168 10.1006/cbir.1993.1052

[B16] Kremer J. R. Mastronarde D. N. McIntosh J. R. (1996). Computer visualization of three-dimensional image data using IMOD. J. Struct. Biol. 116, 71–76. 10.1006/jsbi.1996.0013 8742726

[B17] Li F. Vierstra R. D. (2012). Autophagy: a multifaceted intracellular system for bulk and selective recycling. Trends Plant Sci. 17, 526–537. 10.1016/j.tplants.2012.05.006 22694835

[B18] Lundgren Rose T. Bonneau L. Der C. Marty-Mazars D. Marty F. (2006). Starvation-induced expression of autophagy-related genes in Arabidopsis. Biol. Cell 98, 53–67. 10.1042/BC20040516 16354162

[B19] Makowska K. Oleszczuk S. (2014). Albinism in barley androgenesis. Plant Cell Rep. 33, 385–392. 10.1007/s00299-013-1543-x 24326697 PMC3921450

[B20] Mandaron P. Niogret M. E. Mache R. Monéger F. (1990). *In vitro* protein synthesis in isolated microspores of *Zea mays* at several stages of development. Theor. Appl. Genet. 80, 134–138. 10.1007/BF00224027 24220822

[B21] Maraschin S. F. de Priester W. Spaink H. P. Wang M. (2005). Androgenic switch: an example of plant embryogenesis from the male gametophyte perspective. J. Exp. Bot. 56, 1711–1726. 10.1093/jxb/eri190 15928015

[B22] McDonald K. L. Auer M. (2006). High-pressure freezing, cellular tomography, and structural cell biology. Biotechniques 41, 137–143. 10.2144/000112226 16925014

[B23] Nagl W. (1977). ‘Plastolysomes’ - Plastids involved in the autolysis of the embryo-suspensor in *Phaseolus*. Z. Pflanzenphysiol. 85, 45–51. 10.1016/S0044-328X(77)80263-824306370

[B24] Nitsch C. Nitsch J. P. (1967). Induction of flowering *in vitro* in stem segments of *Plumbago indica* L. I Production of vegetative buds. Planta 72, 355–384. 10.1007/BF00390146 24554330

[B25] Nitsch J. P. Nitsch C. (1969). Haploid plants from pollen grains. Science 163, 85–87. 10.1126/science.163.3862.85 17780179

[B26] Otegui M. S. Noh Y. S. Martinez D. E. Vila Petroff M. G. Staehelin L. A. Amasino R. M.. (2005). Senescence-associated vacuoles with intense proteolytic activity develop in leaves of Arabidopsis and soybean. Plant J. 41, 831–844. 10.1111/j.1365-313X.2005.02346.x 15743448

[B27] Reyes F. C. Chung T. Holding D. Jung R. Vierstra R. Otegui M. S. (2011). Delivery of prolamins to the protein storage vacuole in maize aleurone cells. Plant Cell 23, 769–784. 10.1105/tpc.110.082156 21343414 PMC3077793

[B28] Sangwan R. S. Sangwan-Norreel B. S. (1987). Ultrastructural cytology of plastids in pollen grains of certain androgenic and nonandrogenic plants. Protoplasma 138, 11–22 10.1007/BF01281180

[B29] Satpute G. Long H. Seguí-Simarro J. M. Risueño M. C. Testillano P. S. (2005). Cell architecture during gametophytic and embryogenic microspore development in *Brassica napus*. Acta Physiol. Plant. 27, 665–674 10.1007/s11738-005-0070-y

[B30] Seguí-Simarro J. M. (2010). Androgenesis revisited. Bot. Rev. 76, 377–404 10.1007/s12229-010-9056-6

[B31] Seguí-Simarro J. M. (2015a). High pressure freezing and freeze substitution of *in vivo* and *in vitro* cultured plant samples, in Plant Microtechniques: Methods and Protocols, eds Yeung E. C. T. Stasolla C. Sumner M. J. Huang B. Q. (Cham: Springer Science + Business Media). (in press).

[B32] Seguí-Simarro J. M. (2015b). Three-dimensional imaging for electron microscopy of plastic-embedded plant specimens, in Plant Microtechniques: Methods and Protocols, eds Yeung E. C. T. Stasolla C. Sumner M. J. Huang B. Q. (Cham: Springer Science + Business Media). (in press).

[B33] Seguí-Simarro J. M. Nuez F. (2008). How microspores transform into haploid embryos: changes associated with embryogenesis induction and microspore-derived embryogenesis. Physiol. Plant. 134, 1–12. 10.1111/j.1399-3054.2008.01113.x 18507790

[B34] Shariatpanahi M. E. Bal U. Heberle-Bors E. Touraev A. (2006). Stresses applied for the re-programming of plant microspores towards *in vitro* embryogenesis. Physiol. Plant. 127, 519–534 10.1111/j.1399-3054.2006.00675.x

[B35] Telmer C. A. Newcomb W. Simmonds D. H. (1995). Cellular changes during heat shock induction and embryo development of cultured microspores of *Brassica napus* cv. Topas. Protoplasma 185, 106–112 10.1007/BF01272758

[B36] Testillano P. S. Coronado M. J. Seguí-Simarro J. M. Domenech J. Gonzalez-Melendi P. Raska I.. (2000). Defined nuclear changes accompany the reprogramming of the microspore to embryogenesis. J. Struct. Biol. 129, 223–232. 10.1006/jsbi.2000.4249 10806072

[B37] van Doorn W. G. Kirasak K. Sonong A. Srihiran Y. van Lent J. Ketsa S. (2011). Do plastids in *Dendrobium* cv. Lucky Duan petals function similar to autophagosomes and autolysosomes? Autophagy 7, 584–597. 10.4161/auto.7.6.15099 21460624

[B38] Zaki M. A. Dickinson H. G. (1990). Structural changes during the first divisions of embryos resulting from anther and free microspore culture in *Brassica napus*. Protoplasma 156, 149–162 10.1007/BF01560653

[B39] Zaki M. A. M. Dickinson H. G. (1991). Microspore-derived embryos in *Brassica*: the significance of division symmetry in pollen mitosis I to embryogenic development. Sex. Plant Reprod. 4, 48–55 10.1007/BF00194572

